# Excellence in medical training: developing talent—not sorting it

**DOI:** 10.1007/s40037-021-00678-5

**Published:** 2021-08-20

**Authors:** Gurpreet Dhaliwal, Karen E. Hauer

**Affiliations:** 1 Department of Medicine, University of California San Francisco School of Medicine, San Francisco, CA USA; 2 Medical Service, San Francisco VA Medical Center, San Francisco, CA USA

**Keywords:** Undergraduate medical education, Competency-based education, Medical faculty

## Abstract

Many medical schools have reconsidered or eliminated clerkship grades and honor society memberships. National testing organizations announced plans to eliminate numerical scoring for the United States Medical Licensing Examination Step 1 in favor of pass/fail results. These changes have led some faculty to wonder: “How will we recognize and reward excellence?” Excellence in undergraduate medical education has long been defined by high grades, top test scores, honor society memberships, and publication records. However, this model of learner excellence is misaligned with how students learn or what society values. This accolade-driven view of excellence is perpetuated by assessments that are based on gestalt impressions influenced by similarity between evaluators and students, and assessments that are often restricted to a limited number of traditional skill domains. To achieve a new model of learner excellence that values the trainee’s achievement, growth, and responsiveness to feedback across multiple domains, we must envision a new model of teacher excellence. Such teachers would have a growth mindset toward assessing competencies and learning new competencies. Actualizing true learner excellence will require teachers to change from evaluators who conduct assessments *of* learning to coaches who do assessment *for* learning. Schools will also need to establish policies and structures that foster a culture that supports this change. In this new paradigm, a teacher’s core duty is to develop talent rather than sort it.

**Supplementary Information:**

The online version of this article (10.1007/s40037-021-00678-5) contains supplementary material, which is available to authorized users.

As medical schools have changed approaches to grading and awards (most notably, reconsidering or eliminating honors grades in clerkships [1, 2] and election to honor societies [3]), faculty have raised concerns: How will we recognize and reward excellence? And don’t we *care* about excellence? The decision to change to pass/fail reporting of United States Medical Licensing Examination Step 1 results has further accentuated this concern [4].

Excellence in undergraduate medical education has long been defined by high grades, top test scores, honor society memberships, and publication records. More accolades in relationship to peers has provided a signaling and sorting mechanism for schools and residency programs. This view of excellence is familiar to generations of physicians but is out of sync with the educational experience students deserve and the care that patients need. We propose a revised conceptualization of learner excellence that requires a new model of teacher excellence driven by instructors whose skill is developing talent not sorting it.

## “I know it when I see it”

Faculty convey students’ performance through conversations, evaluations, and letters of recommendation. The most useful narratives describe directly observed skills with examples allowing readers to recognize dimensions of competence. However, many communications are short on details yet feature vague statements of praise and summations such as “top 10% in my career” or “best ever” that reflect a gestalt approach to classifying excellence or an “I know it when I see it” standard.

This pattern recognition approach to characterizing student work parallels pattern recognition in diagnosing illness. Preconditions to trustworthy pattern recognition include frequent exposure to the clinical situation, regular feedback on diagnostic decisions, and continual updates to knowledge about the disease (“illness script” in clinical reasoning parlance) [5]. When learner assessments are made without frequent direct observations of students, without feedback about students’ future performance, and with an outdated “script” of competencies, pattern recognition loses validity.

The traditional script frequently frames excellence along a single dimension (typically, cognitive or technical ability) instead of the multidimensional skills captured in modern competency frameworks. “I know it when I see it” also invites faculty to see what they want to see and invites bias along the way.

## Biased by the familiar

Just as cognitive bias jeopardizes clinical decision-making [6], implicit biases can influence our judgements about learners and predispose teachers to favor some students over others (see Table S1 of the Electronic Supplementary Material). Decades of social psychology research have demonstrated a strong human tendency toward in-group bias, where we positively evaluate or favor our own group (people who resemble us) at the expense of the out-group [7]. Teachers are susceptible to being influenced by concordance (demographic or intellectual) with their learners [8]. We are more likely to see excellence in people who look like us, share our academic pedigree, or excel in areas that we valued during our formative years, which may have been technical proficiency over collaboration or knowledge recitation over skills in learning new content. Grading structures typically reflect these traditional priorities and values [9].

Students with a familiar profile can benefit from teachers’ affinity through subtly upgraded evaluations [10]. Slightly more generous narrative evaluations or scores yield higher grade designations which then open doors to residency programs and medical specialties [11–15]. Students can feel pressure to indicate interest in the same field as their assessors in order to earn favorable evaluations or better learning opportunities [16]. When we are misaligned with students (different backgrounds, beliefs, or prioritized skills) or do not share the same race, ethnicity, or gender [17–19], this cascade works against them. A system focused on categorization that uses gestalt coupled with outdated and biased benchmarking preordains a designation of “excellent” to a few instead of developing excellence for all.

## The educational excellence students and society need

Time spent assessing a student relative to other students (e.g., trying to identify the “best” students) is a poor use of teachers’ abilities. Modern teachers serve students more meaningfully by devoting their energy to fostering each learner’s broad skillset. To do this, teachers need to cultivate their knowledge and skills on topics they may not have formally learned in their training. They must also examine their own ability to interact with increasingly diverse student and patient populations.

For example, students are expected—and are expecting—to become skilled in health advocacy by contributing their expertise and influence to improve the health of different patient populations [20]. This competency includes recognizing health inequities, understanding the needs of communities, speaking on behalf of others when required, and supporting the mobilization of resources to effect change [21]. Teachers cannot rely on their intuition regarding appropriate levels of advocacy. Instead, they must fulfill their commitment to their students by learning what is meant by advocacy, understanding specific milestones that students must meet as they progress in this competency, and seeking opportunities for direct observation [22].

Though advocacy may be new to teachers, assessing a student’s advocacy skills has parallels to assessing a student’s other skills such as doing a lumbar puncture or leading a family meeting. A teacher cannot assess the latter example by saying “I know good communication when I see it.” Instead of using this pattern recognition approach, faculty members must commit to understanding the construct being measured and the specific milestones and subskills that students must achieve as they progress through training [23].

Fostering excellence in advocacy requires faculty to broaden their perspective to incorporate a skillset they may have never considered fundamental to being a physician [24]. This growth process may include practicing perspective taking and openness to patient (and student) life experiences that they never contended with, such as taking multiple buses to an appointment, being denied access or resources based on personal identity, or having to decide between filling a prescription or feeding their family.

When coaching students in advocacy or any other competency, educators must commit to making assessments based on direct observation. Entrustable professional activities are pre-specified workplace tasks (e.g., performing an appendectomy) which allow teachers to observe students integrate multiple competencies in a relevant workplace activity [25]. Teachers who wish to advance their skills in promoting and assessing advocacy would need to prioritize observing a workplace activity such as their student collaborating with a social worker to arrange travel vouchers for a patient. These observations allow the supervisor to identify areas for targeted teaching and growth (e.g., “next time, check with the patient first regarding her preferred time of day for her appointments”). With each data point, the educator must become skilled at making an assessment *for* learning (to drive growth), not an assessment *of* learning (to classify students for an external scheme such as a grade, award, or residency) [26].

Most of us do not “know it when we see it” because we were not trained in an environment when “it” matched the needs of society. New medical curricula now emphasize not only patient advocacy, but also shared decision-making, interprofessional collaboration, social determinants of health, and high-value care. The COVID-19 pandemic highlights the need for teachers with adaptive expertise to train future providers who will be prepared to adapt and learn about emerging health threats and respond using knowledge and skills that may not have existed during their training [27, 28]. The goal of medical education is to develop students who are excellent across these domains, and it will take a new faculty mindset to do that.

## Shifting to a growth mindset

Fostering excellence instead of classifying it entails teachers adopting the same attitude we encourage in learners: shifting from a fixed mindset (“I know excellence in a student when I see it”) to a growth mindset (“I can learn new ways to assess and promote student skill development in unfamiliar domains”) [29]. Schools must undertake several steps to guide faculty into the coaching business and out of the classifying business [30, 31].

Policy changes such as removing honors grade designations and student rankings allow faculty to conduct assessments that are low stakes and formative rather than high stakes and summative [32]. Instead of focusing repeatedly on ill-fated attempts at rater training (getting everyone to evaluate consistently), faculty development should emphasize feedback training (getting everyone to consistently observe, record, and coach) [33]. Training can also engage faculty in examining their own longstanding assumptions and biases [34]. Introducing a new value (e.g., social justice) along with a new role (e.g., coaching) cannot be accomplished through a single training session. It requires frequent communication from leaders, multiple channels of dissemination (e.g., videos, emails, podcasts), and champions within the student body and faculty to effect change gradually and steadily while unequivocally and relentlessly signaling its direction and importance.

Selection of new clinical teachers should emphasize their commitment to directly observing learners’ work and building their own skills to engage learners in feedback discussions [35]. Programs should seek and foster a teacher mindset that welcomes rather than dreads identification of students with weaknesses. Great teachers are not distinguished by their ability to make “top” learners reach even greater heights, but rather by their ability to bring the “not yet” learner onto a developmental trajectory toward competence. The organizational goals must also shift from upholding a reputation for recruiting and producing the “best” graduates toward a culture where improvement and a growth mindset is expected for all individuals and the institution itself [36].

## Competency-based assessment: promising but not a panacea

The framework of competency-based assessment—including specified milestones, developmental trajectories, and direct observation—can guide teachers in their professional evolution. However, the shift to competency-based assessment does not eliminate or solve many long-standing challenges in assessment programs.

The same rater biases outlined earlier that affect summative judgements of performance, including cognitive shortcuts and pattern recognition, can influence what evaluators see and infer in direct observations of learners, particularly those who differ from them. Therefore, teachers who shift from graders to coaches must still educate themselves about these cognitive tendencies and whenever possible, seek countermeasures [37]. While these observations by individual faculty are still judgements [38], emerging literature suggests that the synthesis of multiple subjective assessments, grounded in direct observation of the learner and their work, paints an increasingly accurate picture of a trainee’s competency in the workplace [39]. Schools can mitigate the risks of bias by establishing systems where many evaluators provide input based on detailed observations (not impressions) and by instituting group decision-making—such as a grading or competency committee—where members with diverse backgrounds develop and use a shared mental model of excellence to synthesize data to make a competency assessment [40–42].

Residency programs continue to report challenges with underprepared learners who graduate from medical school [43]. Competency-based assessment will not solve this problem unless the foundation of direct observation is tightly coupled with a plan for improvement and re-assessment. Teachers must commit to making high-quality observations of skills and to an additional step: coaching the student, ensuring that the next supervisor does so, or referring the student to the appropriate resources in the medical school. Teachers must be mindful of the potential to propagate bias based on limited time with a learner (e.g., only one day in clinic or the hospital) and must become skilled at formulating a learner handover for the next supervisor to help the student make progress along their longitudinal trajectory [44]. Schools must establish a centralized reporting system that ensures progress is being made. And for students whose growth is hampered by learner-supervisor discontinuity [45–47], schools must support faculty time and skill development for longitudinal clinical experiences that enable them to coach and mentor.

Teachers must also modify their approach to the traditionally “high achieving” or “high performing” student in a competency-based assessment system. Without a firm commitment to examine all competencies in a milestone-directed way, teachers may fall prey to the halo effect [48]. Once the learner is identified as excellent in one domain (e.g., knowledge as determined by a test score), a teacher may underappreciate or exaggerate the learner’s performance in other domains. These problematic generalizations can lead to other areas (e.g., advocacy or communication) being overlooked or overrated.

As teachers commit to growing their skills in observation and assessing multiple domains, schools must signal to students and faculty that competence across all domains is the foundation of excellence and that improved patient population health and well-being is the objective of these efforts. Teachers and schools must also start preparing themselves to make competency assessments and coaching plans based on data that are connected to patient outcomes. Utilizing measures of performance linked to quality of patient care—e.g., resident-sensitive quality measures [49]—can strengthen educators’ ability to define excellence in service to patients.

## “Improvement” as part of the excellence code

Faculty have a societal obligation to ensure students achieve competence in relevant domains. However, once the threshold of competence is crossed, faculty attention should shift from the degree of accomplishment to the rate of improvement. This means not worrying about whether a student’s knowledge is “excellent” versus “outstanding,” and instead devoting energy to examining the method of improvement each student employs. Learners working to improve must be rigorous in their practice, reflection, and incorporation of feedback [50]. Students who exhibit limited interest in new challenges should warrant greater concern than students who seek clinical cases at the edge of their comfort zone. Excellence can be defined by the learner’s rate of growth, not just their current level of proficiency.

Integrating lifelong learning as a marker of excellence is at odds with current rhetoric where narratives describing “improvement” are code for bad performance [51]. In the new paradigm, assessment of the student’s improvement and commitment to personal growth is a must-have—and the absence of a mention of improvement would be alarming.

## All patients need excellent physicians

In systems with abundant uncategorized data, the brain will always seek simplified abstractions to deal with complexity. Traditional assessment systems fulfill this role for advisors, award committees, and residency programs, and do so in a reductionistic manner based on what academic physicians—not society—value.

The job of medical school is not to sort students for residency, but to develop doctors to meet patients’ and society’s needs [52]. Residencies have the same goal and need not be in the business of sorting for fellowships and clinical practices. We will fall short of this goal as long as we condone the current system that defines excellence using metrics that value trainees who follow narrowly in their predecessors’ footsteps and triage students among residencies and specialties accordingly.

Without categorization by tests, grades, and adjectives, educators anticipate immense difficulty in selecting students for residency programs. This worry reflects the difficulty in selecting residents *as we have always done*, in which traditionally “excellent” students gain entry into “excellent” programs. There is no reason to believe that this sorting system has optimized our workforce to meet societal demands or that it could not be improved upon. Holistic review processes reflect the capacity of schools and residencies to assess excellence across multiple domains and select candidates whose areas of focus, capabilities, approaches to learning, and values match those of the program and society [53–55].

When we employ “I know it when I see it”, we endorse a static version of excellence that is outdated, inaccurate, and exclusionary. The excellence in learners that society needs is a product of teachers who continually grow in their ability to coach and assess across multiple domains. All patients need excellent physicians. It’s our job to develop them.

## Supplementary Information

Table S1. Examples of biases that affect our judgements about other people
