# Supplementary material for: Excellence in medical training: developing talent—not sorting it
Source: Perspect Med Educ. 2021 Aug 20;10(6):356–61. doi: 10.1007/s40037-021-00678-5 (PMC8377327; doi:10.1007/s40037-021-00678-5)
Supplement: Supplementary file 1 — Table S1. Examples of biases that affect our judgements about other people [file 40037_2021_678_MOESM1_ESM.docx]

**Table S1. Examples of biases that affect our judgements about other people**

|  | **Definition** | **Example** |
| --- | --- | --- |
| **Affinity Bias** | Tendency to connect with others who share similar backgrounds, beliefs, and interests | A supervisor extends an extra learning opportunity to the student on the team who attended the same college as they did. |
| **Confirmation Bias** | Tendency to select information that confirms or supports prior beliefs | A supervisor has formed a positive impression of a student’s communication skills based on comments shared informally by a colleague who worked with the student on a previous rotation. When writing the student evaluation on the current rotation, the supervisor incorporates a nurse’s praise of the student’s bedside manner but omits a physical therapist’s concerns about the way the student conveyed bad news. |
| **In-group Bias** | Tendency to be more helpful and positive towards members of our own group than to members of another group (“out-group”) | A supervisor writes supportive evaluations about the advocacy skills of a student who shares the same political beliefs while expressing concerns about the “lack of commitment to patients” of another student with a different political viewpoint. |
| **Performance Bias** | Tendency to assume that members of one group are better at certain tasks than members of other groups based on stereotypes (which leads to members of the dominant group being judged by *expected* potential while individuals in less dominant groups are judged by *current* accomplishments) | A supervisor preferentially invites members of one gender to practice a difficult procedure and then writes evaluations praising those students who practiced and demonstrated skill. For other students who are not afforded those practice opportunities, the supervisor writes that they are “not engaged in nor appropriately developing” that skill. |
| **Status Quo Bias** | Tendency to prefer the current state of affairs and perceive any change from that baseline as a loss | A supervisor enthusiastically recommends a student who demonstrates advanced knowledge about clinical trials and has done biomedical research but is equivocal in their recommendation for a student who demonstrates advanced advocacy skills and is conducting research on health equity. |
